# Surgical treatment approaches and reimbursement costs of surgical site infections post hip arthroplasty in Australia: a retrospective analysis

**DOI:** 10.1186/1472-6963-13-91

**Published:** 2013-03-11

**Authors:** Katharina MD Merollini, Ross W Crawford, Nicholas Graves

**Affiliations:** 1 Institute of Health and Biomedical Innovation, Queensland University of Technology, 60 Musk Avenue, Kelvin Grove, QLD 4059, Australia; 2 Orthopedic Research Unit, Clinical Science Building, Prince Charles Hospital, Brisbane, Australia; 3 The Centre for Healthcare Related Infection Surveillance & Prevention, Queensland Health, Brisbane, Australia

**Keywords:** Hip replacement arthroplasty, Surgical wound infection, Cost analysis, Outcome assessment, Surgical revision

## Abstract

**Background:**

The treatment for deep surgical site infection (SSI) following primary total hip arthroplasty (THA) varies internationally and it is at present unclear which treatment approaches are used in Australia. The aim of this study is to identify current treatment approaches in Queensland, Australia, show success rates and quantify the costs of different treatments.

**Methods:**

Data for patients undergoing primary THA and treatment for infection between January 2006 and December 2009 in Queensland hospitals were extracted from routinely used hospital databases. Records were linked with pathology information to confirm positive organisms. Diagnosis and treatment of infection was determined using ICD-10-AM and ACHI codes, respectively. Treatment costs were estimated based on AR-DRG cost accounting codes assigned to each patient hospital episode.

**Results:**

A total of n=114 patients with deep surgical site infection were identified. The majority of patients (74%) were first treated with debridement, antibiotics and implant retention (DAIR), which was successful in eradicating the infection in 60.3% of patients with an average cost of $13,187. The remaining first treatments were 1-stage revision, successful in 89.7% with average costs of $27,006, and 2-stage revisions, successful in 92.9% of cases with average costs of $42,772. Multiple treatments following ‘failed DAIR’ cost on average $29,560, for failed 1-stage revision were $24,357, for failed 2-stage revision were $70,381 and were $23,805 for excision arthroplasty.

**Conclusions:**

As treatment costs in Australia are high primary prevention is important and the economics of competing treatment choices should be carefully considered. These currently vary greatly across international settings.

## Background

Surgical site infection (SSI) is a complication caused by bacterial contamination of the wound during surgery. SSIs have been reported to occur in 1.5-2.5% of primary total hip arthroplasty (THA) and estimates vary between countries [1]. International treatment practices of deep SSI following THA are also known to differ and strategies to treating these infections in Australia are not well-known. As only small single-centre patient outcomes for a single treatment have been published in this context [2,3] it remains unclear if other treatment approaches are commonly utilised. Understanding current practice and outcomes of deep SSI in Australian hospitals can be useful for policy makers and allows international comparison. Quantifying the economic burden of this adverse event will emphasise the importance of cost-effective infection control in THA and stimulate research into the cost-effectiveness of different treatment decisions.

The Center for Disease Control and Prevention (CDC) distinguishes superficial, deep and joint SSIs [4]. Superficial SSIs involve the patient’s skin and subcutaneous tissue and are typically treated with relatively simple measures, such as antibiotics. Deep SSIs of the deep fascia & muscle layers and joint SSI on the other hand can have devastating consequences. Treatment is resource intensive and places a high burden on health care systems and patients’ well-being [5].

The choice of treatment for deep SSI generally depends on a number of factors, including the type of organism, local factors referring to the bone and tissue condition, the time of infection onset and the patient’s general health status [6]. In the case of early infection onset debridement, antibiotics and implant retention (DAIR) can be a first management strategy [7]. Revisions are a treatment alternative to DAIR and can be performed as one- or two-stage procedures. In one-stage revisions the prosthesis is exchanged in one operation. Two-stage revisions on the other hand are more complex and involve: removing the prosthesis; treating the patient for weeks or months, while they suffer impaired functional ability and quality of life [8]; inserting a new prosthesis once the infection is controlled. A last resort option is to permanently remove the prosthesis in a so-called Girdlestone procedure (excision arthroplasty) [9].

The literature has shown that 2-stage revisions have higher success rates but are typically high cost [5,10]. One-stage revisions and DAIR have lower risks but also have a lower success rate [11].

The objectives of this paper are to describe the frequencies and costs of treatments for deep SSI post primary THA in Australian hospitals and compare the findings with practices reported in international jurisdictions.

## Methods

### Organisation of data

The ‘Queensland Hospital Admitted Patient Data Collection’ (QHAPDC) database includes administrative and clinical data for patients admitted to Queensland hospitals (public/private) [12]. All data were extracted from January 2006 to December 2009 to include diagnosis and treatment codes, gender, admission date, age at admission, date of procedure performed, separation date, mode of separation/discharge status, length of stay, major diagnostic category and Australian Refined Diagnosis Related Groups (AR-DRG) cost codes.

Records were linked with pathology records in ‘Auslab’ to confirm the occurrence and type of infection [13]. Auslab pathology data was classed as relevant if it indicated a positive organism isolated in the patient’s hip. Auslab data was only supplied if a QHAPDC patient record existed.

Ethics clearance was obtained through the Queensland University of Technology Research Ethics Unit (Approval number: 1000000205) and was in compliance with the Helsinki Declaration on research involving human subjects. Formal data access applications were approved by data custodians in Queensland Health and Pathology Queensland.

Patients were selected for analysis if they fulfilled the following three criteria in chronological order:

1) underwent primary THA (defined by ACHI treatment codes 49318–00 or 49319–00)

2) presented with deep SSI, i.e. diagnosed with post procedural wound infection or sepsis following a procedure (defined by ICD-10-AM diagnosis codes T81.41 or T81.42)

3) treated with revision surgery (categorised by ACHI treatment codes) due to deep SSI (defined by Auslab and criteria shown below).

### Criteria used to identify deep/joint SSI

The following criteria were applied to identify deep/joint infections:

✓ Infection occurs within 1 year of primary THA (as per CDC definition)

✓ Treatment of infection was ≥2 months after primary THA (means it is not a superficial SSI as they occur within 30 days, as per CDC definition)

✓ Only deep infections are treated with:

○ Excisional debridement of soft tissue involving bone or cartilage

○ Revision/excision arthroplasty

✓ Auslab specimen collection method / specimen site indicate a deep infection if

○ using an aspirate/ syringe to extract fluid out of hip

○ fluid was collected from the synovial cavity / joint

If the following criteria applied, cases were included as possibly relevant (could be superficial/deep infections):

✓ Auslab specimen collection method was ‘swab’ or ‘tissue’

✓ Excisional debridement of soft tissue

As Auslab records did not reliably record whether the infection was deep the criteria outline above were applied to select deep infections in the data set. Relevant treatment strategies include debridement, antibiotics & implant retention (DAIR), 1-stage revision, 2-stage revision and excision (ACHI treatment codes used for each category is available from authors on request).

### Analysis

The frequencies of different treatment strategies were estimated over time. Treatment was classed as successful if no further surgery was undertaken due to deep SSI within one year post primary THA.

Costs were derived from treatment codes and related costs reported in the National Hospital Cost Data Collection [14] for each hospital episode. The average cost of each treatment pathway was based on the number of cases in the dataset and average total costs as per AR-DRG. For patients who underwent primary THA and SSI treatment in one hospital episode (8%) only costs exceeding the average costs of a standard primary THA (AR-DRG code I03C: $18,897) were attributed to the particular treatment. This is based on the assumption that costs for primary THA would occur in any case and hence only infection treatment costs were in addition to normal costs.

The total average cost of one deep SSI represents the average treatment costs per patient across all treatment strategies.

## Results

### Patient descriptives

Patient characteristics are described in Table 1. There were 10,874 records of patients undergoing primary THA with n=114 (1.05%) individual patients requiring further hospital care due to SSI (n=75 definitely deep, n=39 possibly deep). In this group there were slightly more females (53.5%) and the majority of patients were aged 60–79 years (65.8%).

**Table 1:** Characteristics of n=114 patients with deep SSI in the final dataset

**Patient outcomes**	**N**	**%**
**Total, patients**	**114**	**100**
**Gender**		
female	61	53.5
male	53	46.5
**Age groups**		
35-49	5	4.4
50-59	20	17.5
60-69	38	33.3
70-79	37	32.5
80-85+	14	12.3
**Classification of infection**		
Possibly deep infection	39	34.2
Deep/joint infection	75	65.8

### Treatment over time

A total of n=114 patients were assessed regarding their first treatment for deep SSI since primary THA. The majority of these patients (74%) experienced early infection onset (≤2 months) which was typically managed with DAIR. Delayed infections (occurring within 3–12 months) were most frequently treated with 1-stage revisions (10%), but also DAIR (8%) and 2-stage revisions (7%). Permanent removal of the prosthesis (excision) was rarely utilised as first treatment option.

### Treatment outcomes & related costs

The majority of patients (59.6%) underwent DAIR as first treatment which was successful in eradicating the infection in 60.3% (n=41) of patients, incurring average costs of $13,187 (range: $5,565-$36,408). A total of 39.7% (n=27) of patients first treated with DAIR required multiple treatments, associated with average total costs of $29,560 (range: $14,566-$63,069) (see Table 2).

**Table 2:** DAIR treatment failure with subsequent treatment (n=27)

**Treatment sequence†**	**N (%)**	**Average cost (AUD$)**	**% of total cost**	**Total average cost (AUD$)**
2x DAIR	11 (40.7%)	23,026	31.7%	**29,560**
1x DAIR, 1-stage Revision	5 (18.5%)	31,769	19.9%	
3x DAIR	3 (11.1%)	25,708	9.7%	
1x DAIR, 1-stage Revision, 1x DAIR	2 (7.4%)	63,069	15.8%	
1x DAIR, 2-stage Revision	1 (3.7%)	39,028	4.9%	
1x DAIR, Excision	1 (3.7%)	23,973	3.0%	
2x DAIR, Excision	1 (3.7%)	23,477	2.9%	
3x DAIR, Excision	1 (3.7%)	34,698	4.3%	
4x DAIR, Excision, 1x DAIR	1 (3.7%)	47,000	5.9%	
8x DAIR	1 (3.7%)	14,566	1.8%	
Total	27 (100%)	798,132	100%	

†DAIR = Debridement, antibiotics and implant retention.

One-stage revision was the initial treatment for 25.4% (n=26) of patients and resulted in an 89.7% success rate, costing on average $27,006 (range: $8,957-$36,408). These costs were based on n=25 patients; 1 patient was excluded to avoid an overstatement of costs as multiple treatments were performed within the same hospital episode (only one cost code was assigned for related and unrelated treatment). For 10.3% of patients the initial 1-stage revision failed and patients subsequently underwent DAIR (66.7%) or excision arthroplasty (33.3%). The average cost of these multiple treatments was $24,357 (range: $15,801-$36,408) (see Table 3).

**Table 3:** One-stage revision treatment failure with subsequent treatment (n=3)

**Treatment sequence†**	**N (%)**	**Average cost (AUD$)**	**% of total cost**	**Total average cost (AUD$)**
1-stage Revision, Excision	1 (33.3%)	15,801	21.6%	**24,357**
1-stage Revision, 1x DAIR	1 (33.3%)	20,862	28.6%	
1-stage Revision, 3x DAIR	1 (33.3%)	36,408	49.8%	
Total	3 (100%)	73,071	100%	

†DAIR = Debridement, antibiotics and implant retention.

Two-stage revision was the first treatment in 12.3% (n=14) of patients and showed a success rate of 92.9% with average costs of $42,772 (range $15,801-$60,870). One patient (7.1%) required subsequent treatment was a 1-stage revision to eradicate the infection, costing $70,381 for both these procedures (see Table 4).

**Table 4:** Two-stage revision treatment failure with subsequent treatment (n=1)

**Treatment sequence**	**N (%)**	**Average cost (AUD$)**	**% of total cost**	**Total average cost (AUD$)**
2-stage revision, 1-stage revision	1 (100%)	70,381	100%	**70,381**
Total	1 (100%)	70,381	100%	

Excision arthroplasty was the primary treatment in 2.6% (n=3) of cases, all of which were followed by multiple DAIR procedures. This treatment pathway resulted in total average costs of $23,805 (range: $23,477-$24,462) (see Table 5).

**Table 5:** Excision treatment failure with subsequent treatment (n=3)

**Treatment sequence†**	**N (%)**	**Average cost (AUD$)**	**% of total cost**	**Total average cost (AUD$)**
Excision, 2x DAIR	2 (66.7%)	23,477	65.7%	**23,805**
Excision, 3x DAIR	1 (33.3%)	24,462	34.3%	
Total	3 (100%)	71,416	100%	

†DAIR = Debridement, antibiotics and implant retention.

Overall, about one third (32.5%) of deep SSI patients underwent multiple surgical procedures. The total average treatment cost per deep SSI was $24,644 across all treatments or overall $19,688 for patients with DAIR as 1^st^ treatment (success/failure), $26,722 for 1-stage revision, $44,744 for 2-stage revision and $23,805 for excision arthroplasty.

An overview of organisms identified in pathology records is given in Table 6.

**Table 6:** Organisms identified in pathology (Auslab) confirmed infections

**Organisms identified (total)**	**N (250)**	**% (100)**
Staphylococcus	119	47.6
Coagulase	23	9.2
Enterococccus	23	9.2
Enterobacter	17	6.8
Pseudomonas	17	6.8
Escherichia coli	15	6.0
Candida albicans	8	3.2
Klebsiella	8	3.2
Serratia	8	3.2
Other	11	4.4

## Discussion

### Australian context

In this study 114 patients required 178 separate surgical procedures to control the infection. The majority of these patients had an early onset of deep SSI (74%). DAIR was most commonly the first choice of treatment for deep SSI (59.6%) and resulted in the lowest total average cost of $19,688 when all outcomes and further treatments were considered. One-stage revision was the next most frequent first treatment (25.4%) with average costs of $26,722. Two-stage revisions and excision arthroplasty were less commonly chosen as primary treatment option (12.3% and 2.6%, respectively) and led to costs of $44,744 and $23,805 respectively. Treatment costs of SSI following THA in Australia were previously estimated by Smith et al. [15] based on patient data from the Canberra Hospital (ACT). The average cost of deep SSI treated with 2-stage revision was $79,623. The cost discrepancy compared to our estimate may be due to technological changes, differences in patient co-morbidities or severity of infection.

### Study limitations

We defined deep SSI as per CDC definition as occurring within one year of primary THA [4] and did not include late infections. We attempted to select all deep SSI in the data but 39 indistinguishable cases were included in the analysis. As these patients may have been treated for superficial SSI, the proportion of treatment with DAIR might be inflated and underestimate treatment costs.

The fact that our cost calculations were based on AR-DRG codes is likely to further understate true costs. The DRGs reimbursement system has been criticised for understating costs of treating prosthetic joint infections and resulting in financial losses for treatment centres [16,17]. These cost codes estimate only direct in-hospital costs (ward medical/nursing, non clinical salaries, pathology, imaging, allied, pharmacy, critical care, operating rooms, emergency department costs, supplies, special suites, prosthesis, oncost, hotel costs, depreciation and overhead costs) [14]. For comprehensive cost estimates all direct post-discharge costs (e.g. antibiotics, primary care services, travel costs, other pharmaceuticals) and indirect costs would have to be considered which was not feasible within this work. Examples of indirect costs would be production losses for patients, damage to clinicians’ reputation and the real economic opportunity cost is the loss arising from blocked hospital beds or resources due to SSI treatment that cannot be utilised for other purposes [18].

The overall percentage of deep SSI picked up by our data was estimated at 1.05%. This might not reflect the true incidence but is consistent with previous estimates [19]. Infections as reason for revision may be under-diagnosed as other conditions caused by infection can be selected as principal reason for revision, such as loosening or lysis. Furthermore, our dataset was not designed to capture follow-up data of patients undergoing primary THA at the end of our selected time period which might further underestimate true infection rates.

### International comparison

The choice of first treatment varies internationally. A recent retrospective cohort analysis [20] conducted in Switzerland recorded the following treatments for 68 patients with prosthetic joint infection of the hip/knee: 75% had 2-stage revision, 17.6% DAIR, 5.9% 1-stage revision and 1.5% resection or suppressive antibiotic treatment. This suggests a high cost response to the problem, that is likely to have good success rates but patients will have endured the hardship of two stage treatments.

A Taiwanese study [21] of 53 prosthetic joint infection patients reported the most common treatment strategy to be DAIR (51%), followed by 2-stage revision (30%) and resection arthroplasty (19%). It becomes evident that these settings have a higher proportion of 2-stage revisions rather than 1-stage revisions.

Two-stage revisions used to be the gold standard in treating deep SSI/prosthetic joint infections and still seem to be the preferred choice in some countries, including the United States, Czechoslovakia and Switzerland [1,22,23]. But preferences vary from centre to centre and a generalisation of treatment patterns is difficult.

rOur study showed that treatment with 2-stage revision esulted in a higher success rate (92.9%) compared to 1-stage revision (89.7%) which is in line with findings from the literature [10,22]. Recent studies from Austria and New Zealand have argued that the fear of re-infection should not dominate the choice of treatment and that 1-stage revisions should be favoured as they result in better functional outcomes for patients whilst reducing mortality [24,25]. Furthermore, inadequate reimbursement of in-hospital treatment costs may encourage German facilities to undertake 2-stage revisions rather than 1-stage revisions in order to minimise their economic loss [17].

There is a trend towards 1-stage revisions which should be the preferred treatment as long as no difficult-to-treat organisms are present (e.g. MRSA). Our data furthermore showed that if the first treatment with DAIR was unsuccessful repeated DAIR showed good results when the unclear cases of SSI were included (success rate of second DAIR was 61%).

### The larger challenge

Choosing the right treatment is a complex task and the costs and health benefits arising from different approaches should guide surgical decision making. A technically efficient health service should achieve the maximum health benefits from scarce resources and the costs and benefits incurred by providers and patients are relevant. Estimating these is complicated by patient heterogeneity and the different treatment algorithms that have been proposed, considering various factors, such as the time of infection onset, type of organism and surgeon experience [6,7].

The priority of orthopaedic surgeons is to achieve the best clinical outcomes for each individual patient. In other words, to maximise the quality of life whilst minimising mortality, often expressed as a single outcome measure called quality adjusted life years (QALYs). At the same time, health care facilities face economic pressures and attempt to minimise treatment costs. Therefore, they should attempt to provide the best value for money treatment in terms of health benefits (QALYs gained). There may be a tension between the needs of the patient and surgeon and the resources available. This tension might explain the different treatment patterns observed in different settings with weaker or stronger central control of how scarce resources are used.

If only cost outcomes were important DAIR would be selected as the initial treatment for all patients as it generated the lowest average costs (although costs may be understated due to inclusion of potential superficial SSI cases). Costs for our cohort of 114 patients would decrease by around $5,000/patient, suggesting cost savings of $570,000. Nevertheless, this simplified calculation disregards underlying factors, such as the presence of resistant organisms or late infection onset which usually require treatment with 2-stage revision. It also does not take into account that DAIR is associated with lower success rates resulting in more patients having to undergo multiple surgical procedures, each associated with surgical mortality risks and decreased quality of life.

An adequate evaluation of the cost-effectiveness of different treatment algorithms implies a large amount of data. Information would be required for each patient on the total costs of treating them with each alternative and the expected health benefits in QALYs. A micro-simulation model might be required that tracks each patient as an individual, rather than testing alternatives on a hypothetical cohort of individuals. Due to patient heterogeneity, the outcomes of each patient associated with each treatment alternative would have to be identified to allow comparisons of cost-effectiveness. Many clinical factors impacting on the outcomes are not recorded routinely and are rather based on judgements by experienced surgeons.

A recent decision analysis by Wolf et al. [26] compared direct exchange and two-stage revision as treatment for infected THA in terms of QALYs gained. They found that direct-exchange arthroplasty resulted in better health outcomes despite the higher re-infection risk.

Similarly, Fisman et al. [27] published the results of their decision model for hypothetical patient cohorts to investigate the management of infected THA using either DAIR or 2-stage revision. They found that DAIR resulted in an increased life expectancy (2.2-2.3 quality-adjusted life months) and an improved cost-effectiveness ratio. The authors recommended this treatment for the US context, given the presence of easy-to-treat organisms and non-loosened prosthesis.

Although these results are useful one should be cautious about drawing conclusions for Australian health care facilities. Beside differences in health care systems and likely differences in treatment costs, a major limitation is that treatment patterns differ as our data revealed that 1-stage revisions are favoured over 2-stage revisions. Future studies should also consider all relevant treatment pathways (including multiple treatments) and if possible account for patient heterogeneity by using a micro-simulation approach.

To date, no research has addressed the major and difficult-to-answer question of the ideal treatment for an individual patient from the health services perspective, i.e. the treatment with the lowest cost of extra health gains. The costs of individual treatment pathways identified in this work might be a first step in providing decision makers with more information on the cost-effectiveness of treatment alternatives.

Primary prevention could decrease a large number of SSI following THA. Relevant infection prevention strategies should be explored and their cost-effectiveness analysed. Synthesised information on the effectiveness of strategies as well as health outcomes is required for a thorough evaluation and would contribute to decreasing the economic burden of this adverse event following hip arthroplasty.

## Conclusions

This paper has contributed to a better understanding of the treatment and economic consequences of deep SSI treatment following primary THA in Australia. It is important to recognise that only accounting for AR-DRG costs is likely to understate true costs of treating deep SSI which are still a major burden for patients and health care facilities.

All efforts should be made to provide patients with the best possible health outcomes whilst managing infections in a cost-effective way. Although DAIR has been suggested in this context for American hospitals, these results may not be generalisable to the Australian setting and had limitations. A micro-simulation based on individual patients and a range of clinical factors may be required for reliable predictions.

The importance of primary prevention should not be neglected even if the incidence of deep SSI is unlikely to be zero. The occurrence of SSI might be reduced by introducing more cost-effective infection prevention measures. This would also reduce the economic burden of SSI to healthcare services in the long-term and improve patient outcomes.

## Abbreviations

AR-DRG: Australian refined diagnosis related group; CDC: Center for disease control and prevention; DAIR: Debridement, antibiotics and implant retention; QALY: Quality adjusted life year; QHAPDC: Queensland hospital admitted patient data collection; SSI: Surgical site infection; THA: Total hip arthroplasty.

## Competing interests

All authors report no conflicts of interest relevant to this article.

## Authors’ contributions

KM designed and conducted the study and drafted the manuscript. RC made substantial contributions to the clinical definition and interpretation of data. NG assisted with the design, coordination and drafting of the manuscript. All authors read and approved the final manuscript.

## Pre-publication history

The pre-publication history for this paper can be accessed here:

http://www.biomedcentral.com/1472-6963/13/91/prepub
